# The role of disclosure in relation to assent to participate in HIV-related research among HIV-infected youth: a formative study

**DOI:** 10.1186/1758-2652-12-17

**Published:** 2009-08-27

**Authors:** Amy Corneli, Lara Vaz, Jennyfer Dulyx, Serge Omba, Stuart Rennie, Frieda Behets

**Affiliations:** 1 Department of Epidemiology, University of North Carolina at Chapel Hill, USA; 2 Department of Health Behavior & Health Education, University of North Carolina at Chapel Hill, USA; 3 School of Public Health, University of Kinshasa, Kinshasa, Democratic Republic of Congo; 4 Department of Dental Ecology, University of North Carolina at Chapel Hill, USA; 5 Department of Social Medicine, University of North Carolina at Chapel Hill, USA

## Abstract

**Background:**

The objective of this study was to develop a culturally appropriate approach for obtaining assent from children aged eight to 17 years to participate in paediatric HIV-related operational research in Kinshasa, Democratic Republic of Congo (DRC). Included within this objective was to determine whether or not HIV disclosure should be included as part of the assent process prior to research participation, a component of research participation, or not incorporated in any aspect of the child's involvement in the research. Factors that influence parents' and caregivers' decisions to disclose HIV status to children in non-research contexts were also explored.

**Methods:**

A qualitative formative study was conducted. Semi-structured interviews were conducted with 19 youth living with HIV, 36 parents and caregivers of youth living with HIV, and 17 health professionals who provide care and support to youth living with HIV and their families. Participants were purposefully selected from three HIV care, treatment and/or psychosocial support programmes in Kinshasa, DRC.

**Results:**

Most youth interviewed believed minors participating in HIV-related research should be informed of their HIV-positive status. Parents and caregivers and health professionals had varied perspectives on if and when HIV status should be disclosed to minors during research participation. The age of the youth influenced parents and caregivers' responses, and disclosure to adolescents was more frequently supported than disclosure to children.

Several parents and caregivers, as well as some health professionals, suggested that minors should never be told their HIV-positive status when participating in HIV-related research, regardless of their age. Within the context of treatment programmes, disclosure of HIV status to minors was supported by youth, parents and caregivers, and health professionals as a means to improve adherence to medication.

**Conclusion:**

In settings where most minors are unaware of their HIV infection, researchers should consider excluding the term, "HIV", when explaining HIV-related research to minors, and omitting it from assent forms or informational sheets related to research participation. However, an individualized disclosure plan should be initiated with parents and caregivers at the time of enrolment in HIV-related research, particularly in research that involves treatment.

## Background

Within the context of operational research, we initiated a paediatric HIV care programme that included free access to antiretroviral treatment in Kinshasa, the Democratic Republic of the Congo (DRC), the first of its kind in the country. The aim of this operational research was to develop a context-appropriate model of comprehensive HIV care and treatment.

During the ethics review of the operational research, it was recommended to obtain assent from children participating in the research, if culturally appropriate. No national guidelines were available in the DRC for assent procedures or disclosure of HIV status to HIV-infected children, and local clinicians reported that few HIV-infected minors in Kinshasa had been informed of their HIV status.

Additionally, at that time, very little was known in the sub-Saharan African context about what role, if any, children play in decision making on research participation, and if, when and how children ought to be told they have HIV. Further, research on the impact of inadvertent disclosure prior to or during research participation was not well studied.

In searching for other guidelines on paediatric HIV disclosure, we learned that few guidelines exist; only US guidelines were found and they focus on US populations. The American Academy of Pediatrics (AAP) recommends the disclosure of HIV diagnosis to children and adolescents [1]. For younger children, AAP recommends partial disclosure that involves a discussion regarding the illness, but the child's diagnosis need not be disclosed. For school-aged children, disclosure is recommended, although a child's knowledge and coping ability should be assessed prior to disclosure, and systems should be in place to help the child cope with the diagnosis. AAP recommends that an HIV diagnosis be disclosed to adolescents.

As for assent guidelines, according to US regulations [2], children should provide assent to participate in research taking into consideration age, risks, benefits and context.

In HIV-related research with HIV-infected minors, one could argue that minors must be aware of their HIV status, and that HIV must be mentioned in the assent form in order for the assent to be meaningful. In a recent commentary, Barfield and Kane [3] raise awareness of issues surrounding meaningful assent from HIV-infected youth, and suggest further dialogue on the delicate question of how to balance disclosure and assent.

For our operational research, we questioned whether minors should know the reason they would be receiving care and treatment in order for the assent to be genuinely informed. However, given the local context in Kinshasa of limited disclosure to minors, we had serious concerns that mentioning HIV on the assent form would disclose HIV status to minors and possibly subject them to social and psychological harm, given widespread HIV-related stigma and discrimination in the local context.

For these reasons, we conducted a formative study from July to September 2005 to determine the best assent approach for our operational research. The objective of the formative study was to develop a culturally appropriate approach for obtaining assent from children aged eight to 17 years to participate in the operational research; the legal age of consent in DRC is 18 years of age.

In pursuit of this objective, we explored whether or not HIV disclosure should be included as a part of the assent process prior to research participation, a component of research participation, or not incorporated in any aspect of the child's involvement in the research. We also explored disclosure experiences among youth and parents and caregivers in non-research settings, as well as culturally appropriate approaches for disclosing an HIV diagnosis to minors as this information could inform our assent procedures or procedures to disclose during participation in the operational research.

While waiting for formative study results, we took a conservative approach to informing and obtaining assent from children who participated in the operational research, which was approved by all affiliated institutional review boards. We obtained written informed assent from minors aged 13 to 17 years, and read an informational sheet to children aged eight to 12 years; HIV was not mentioned to the minors or children in either situation. Parental permission was obtained; HIV was mentioned during the permission process.

## Methods

Semi-structured interviews were conducted with: youth living with HIV, aged 11 to 21 years, who had been previously informed of their HIV status; parents and caregivers of youth who had been informed of their HIV status; parents and caregivers of youth who had NOT been informed of their HIV status; and health professionals who provide care and social support to minors with HIV and their families.

The age eligibility criterion for minors was from eight to 17 years of age; however, the youngest minor to participate was 11 years old. Additionally, young adults aged 18 to 21 years old were included in our sample because advice received from local staff during protocol development indicated that it may be difficult to find an adequate sample size of minors aged eight to 17 who were previously informed of their HIV-positive status. Hence, young adults aged 18 to 21, who were disclosed to when they were minors, were recruited and the interviews focused on their disclosure experiences as minors. In this paper, we refer to both minors and young adults as "youth".

Interviews with youth explored the disclosure experience, positive and negative outcomes of disclosure, research decision making, and perceptions of informed assent in relation to HIV research in which participants are recruited from health facilities. Interviews with parents and caregivers of youth who had been informed of their HIV status explored decisions by parents and caregivers on the timing of disclosure of the youth's HIV-positive status to the youth in non-research contexts, factors influencing this decision, and anticipated and actual positive and negative consequences of disclosure.

Parents and caregivers of youth who had not been informed of their HIV status were asked questions about the reasons for non-disclosure, and if and when they were considering disclosure. A separate group of parents and caregivers were asked questions related to assent for HIV-related research in which participants are recruited from health facilities, such as their perceptions of the role minors should play in decision making on research participation, and when minors should be told of their HIV-positive status when participating in such research.

Health professionals were asked to discuss their experiences in disclosing to children their HIV-positive status, as well as questions related to timing of disclosure within a research setting and in general. We did not describe the current assent approach used for the operational research in any of the interviews; topics were explored independently. Interview guides were informed by previous research on disclosure and on decision making in health care and research [4-8].

All participants were purposefully selected and recruited from three centres that provide care, treatment, and/or psychosocial support services to HIV-infected children and their families, including our care and treatment programme. Disclosure of HIV status to minors was not a requirement at any of these facilities for minors to receive services. Centre staff identified youth who had previously been told their HIV status based on their knowledge of working with the youth and centre records.

Interviews were not conducted with youth who could not state they had HIV, AIDS or any other local term for HIV/AIDS during the screening process. Few children were told their HIV status in this setting, and this study interviewed all youth that centre staff could identify that met the inclusion and exclusion criteria, and who were willing to participate.

The primary parent or caregiver of HIV-infected youth who were receiving services at one of the three centres was recruited; parents and caregivers also had to know their children's HIV status to participate in the interviews. The latter criterion was included since it was possible that only one parent or caregiver was aware of the youth's HIV-positive status. The criterion therefore was used to avoid any inadvertent disclosure of the child's HIV status to a secondary caregiver who accompanied the child to the clinic, but who did not know the youth's HIV-positive status. The status of disclosure to the child determined the eligibility of parents and caregivers to be interviewed on either disclosure or nondisclosure, but not on assent.

Six trained Congolese interviewers conducted interviews in either French or Lingala, the local language. Immediately following each interview, interviewers simultaneously transcribed and translated their interview from Lingala into French, or transcribed the interview if originally conducted in French. One of four translators translated the French transcripts into English; two bilingual analysts verified the translations by comparing, word-for-word, the English and French versions.

Data were analyzed using qualitative content analysis. Deductive codes were developed first, and were typically associated with one or two questions on either assent or disclosure from the questionnaires. Inductive codes were developed after reading the initial transcripts. All codes were then applied to the data by at least two analysts using Atlas-TI v. 5.2. Inter-coder reliability involved two analysts coding selected transcripts separately, comparing codes, and then resolving discrepancies when necessary. Inter-coder reliability steps continued until the analyst pairs reached approximately 90% agreement. Each code report was summarized using data display and reduction tables, developed in analyst pairs, and the major themes within each code were identified.

### Ethical approvals

The formative research was approved by the Institutional Review Boards at the Kinshasa School of Public Health in the DRC and at the University of North Carolina at Chapel Hill in the US. All participants in the formative research provided their written informed consent (18 years and older), written informed assent (13–17 years), or were read an informational sheet (8–12 years). Parental permission was also obtained.

## Results

We present here an overview of the data on assent and disclosure, focusing on the preferences and timing of disclosure for minors participating in research when they are unaware of their HIV status. Some findings on disclosure experiences are also described to provide a broader context of disclosure in this setting as well as to provide relevant information for developing recommendations on disclosure and assent for HIV-related research among HIV-related youth. Detailed findings on experiences with disclosure are presented elsewhere [9].

### Sample size and demographics

Table 1 lists all study participants by interview group and topics discussed. Table 2 provides demographic information for the parents and caregivers, youth and health professionals whose responses are presented here.

**Table 1:** Number of participants and overall topics discussed

Participant	N	Topic(s)
Youth living with HIV	19	Assent and disclosure

Parents and caregivers whose child knew her or his HIV status	21; 18 were the primary caregivers of the 19 youth interviewed; 3 were caregivers of youth not interviewed	Disclosure

Parents and caregivers whose child did not know her or his HIV status*	20	Disclosure

Parents and caregivers of a HIV-positive youth; disclosure status of youth not a factor for selection**	15	Assent

Health professionals	17	Assent and disclosure

*Data not presented here

**Based on recruitment records, most, if not all, of these youth had not been disclosed their HIV-positive status

**Table 2:** Demographic information

Characteristics of parents and caregivers interviewed		Parents and caregivers of youth who had been informed of her or his HIV status, interviews on disclosure (n = 21)	Parents and caregivers, interviews on assent (n = 15)
Gender	Female	81%	73%

Age	Median [Range]	45 [25–75]	47 [27–72]

Marital Status	Single	5%	27%
	
	Formally married or living with partner	48%	47%
	
	Widow or widower	43%	20%
	
	Divorced	5%	6%

Education	None	19%	--
	
	Completed some or all primary school	19%	13%
	
	Completed some or all secondary school	43%	74%
	
	Completed some post-secondary school	19%	13%

Relationship to child	Biological mother	43%	33%
	
	Biological father	14%	20%
	
	Grandparent	14%	13%
	
	Aunt or uncle	19%	33%
	
	Sibling	10%	--

Age of HIV-positive child	Median [range]	17 [11–21] (21)	12 [8–18]

**Characteristics of youth interviewed**			**n = 19**

Gender	Female		58%

Age	Median [range]		16 [11–21]

Education	Attending school [age group]		50% [<18] 20% [18–21]

**Characteristics of health professionals interviewed**			**n = 17**

Gender	Female		53%

Age	Median [range]		43 (30–58)

Profession	Clinician		35%
	
	Social assistant		47%
	
	Other		18%

### HIV disclosure in general

#### Youth responses

Interviews with youth revealed that they believed minors want to be told their HIV-positive status when participating in HIV-related research, and also outside of the research context. Most youth interviewed believed it was better to have been informed of their HIV-positive status than to not know.

Common throughout the interviews, youth stated that they believed minors would want to know what they have been suffering from. Youth also expressed that once minors know their HIV-positive status, they can protect themselves, as well as not transmit the illness to others. Additionally, interviews with youths revealed a strong link between disclosure and their reported adherence to medication regimens, as many youth who were interviewed were taking antiretrovirals or other HIV-related treatment. Many youth indicated that knowing their HIV status helped them to take their medicines regularly.

For example, a 13 year-old girl said:

Before I was refusing to take my medicine, but since they announced to me that I was infected with that disease, I started taking medicines without any problems.

An 11 year-old girl said:

It's in order to have it in my mind [knowing her HIV status], to know that I have to take medicines. Because it might happen that Mother forgets to give me the medicines or that she is absent from home, but I myself can look where they put the medicine bottle, if there are some tablets to be taken at that moment. I can take them all by myself.

#### Parent and caregiver responses

As Vaz *et al *(2008) describe in more detail [9], parents and caregivers of youth who knew their HIV status (n = 21) were asked the age when their children were first informed of their HIV-positive status. Ages ranged from 10 to 18 years of age with a median age of 15. Most minors were disclosed to within one year of diagnosis and were adolescents at the time of disclosure.

Only one parent/caregiver mentioned regretting the decision to disclose to her or his child. Many parents and caregivers reported, however, that they continued to sustain feelings of worry or concern about their child's HIV-positive status. For most of these parents and caregivers, their concern was about the illness itself and not due to the fact that their child was now aware of her or his HIV-positive status.

Several parents and caregivers reported feeling relieved or happy with their decision. A few parents and caregivers also described similar observations regarding the association between disclosure and adherence as reported by youth. Several parents and caregivers mentioned that a reason they chose to disclose was to improve treatment adherence or because treatment had become available; some described their perception that improved adherence resulted from disclosure.

For example, a biological mother of a 17-year-old girl said:

[The announcement] has had a great impact on her way of taking medicine. She swallows them without any problem, which is different from the past. She is conscious that her health is dependent on those tablets.

Other reasons for telling the youth were also influenced by the age and health status of the child.

#### Health professional responses

Most health professionals believed disclosure would lead to better management of the youth's illness. Moreover, many health professionals described that, in their experience, one of the reasons parents and caregivers disclose is to improve their child's adherence to medication.

One health professional, a female nurse counsellor, explained:

There are very alert children who no longer want to take medicines when they are in good health. They ask a lot of questions, so the parents look for how to get them to take the medicines.

### Disclosure and participation in HIV-related research

#### Youth responses

When asked whether or not it is acceptable to enrol minors in HIV-related research without the minors' knowledge of their HIV positive status, only three of the 18 youth who answered this question said youth did not need to be told their HIV-positive status when participating in HIV-related research; most youth believed it was unacceptable to enrol minors in such research without disclosure. The most common reason provided was that minors had a right to know the name of the disease that affects them.

For example, an 18-year-old boy said:

After all, it's his disease. Why do you want to hide it from him?

A 16-year-old girl said:

I should know my illness. Why would they hide the name of my illness from me?

Other youth said that minors should know their status so they would not be surprised when hearing their status during the study or have difficulties in answering study questionnaires.

A 17-year-old girl explained:

If they become aware that they suffer from that disease during their participation, this will make them feel uneasy. They will say to themselves, "How are they aware of my disease, while I am not myself informed about it?" It is better for them to know it in advance, before their participation in the research study. They must also know the reasons of their participation in the research study – what they will talk about in the study – so that they can give answers to all the questions that will be asked.

A few youth said that minors would become upset with their parents or caregivers if they were not told their HIV-positive status before enrolling in HIV-related research.

For example, another 18-year-old boy said:

Imagine that you come to participate in a research study on your disease but your parents haven't told you that you have this disease. First, you won't be able to answer questions well because you do not know that you suffer from that disease. [Second] it might [cause problems] at home because your parents hid it from you.

Of the three youth who believed that minors need not be told their HIV-positive status prior to research participation, two believed that minors would be sad or disappointed when learning the news, or the minor's health status would worsen upon learning her or his status. The other participant believed that the minor would tell his friends, which would result in his friends using witchcraft against him.

#### Parent and caregiver responses

Parents and caregivers who were interviewed on issues related to assent (n = 15) were asked if minors should be enrolled into HIV-related research without knowing their HIV-positive status. Questions were asked about children and about adolescents to explore any potential differences in disclosure based on the age of the minor. Minors were first described to interviewees as children under 18 years of age. Parents and caregivers were then asked to define the age in which they perceived children to become adolescents. Ages given ranged from 11 to 15 years.

Parents and caregivers varied in their beliefs about if and when HIV status should be disclosed to minors during research participation. While approximately one-third of parents and caregivers said it was wrong to enroll HIV-infected minors in HIV-related research without the minor knowing she or he had HIV, more parents and caregivers felt it was acceptable. The most common reason provided was that the research could prepare the minor to learn her or his status.

For example, an aunt and primary caregiver of a 12-year-old girl said:

[This would be acceptable] because her [participation in the research] can serve to prepare her to come to know her state.

Conversely, among the parents and caregivers who said it would not be acceptable to enroll minors into HIV-related research without prior knowledge of their status, one stressed that children should be informed prior to research participation because children should be aware of their health status and also because it would improve their ability to be a helpful study participant.

According to this participant, a biological mother of a 15-year-old girl:

It is simply not good, she must be conscious of who she is and what she represents. It is like what I told you before – how can a job be well done if you don't know how to do it and what is it for?"

All parents and caregivers who were interviewed on issues related to assent were asked whether they believed that minors who participate in HIV-related research should be told their HIV status before being enrolled in a study, during the enrolment into a study, during the study, or whether the timing should not be tied to research participation at all; youth were not asked this question. Seven parents and caregivers said children should not be told, and four said adolescents should not be told at all during research participation. These parents and caregivers emphasized perceived distressing effects of disclosure on children. Often, these parents and caregivers said disclosure should not take place until children were older.

For example, a biological mother of an eight-year-old girl said:

They don't have to be told. You have to let them grow up to a certain age. They will have to be told when they have a sense of understanding and wisdom ...giving such information to a child can be fatal to him and at that moment, then, what will be the use of the study?

Eight parents and caregivers said that children should be told at some point during participation in HIV-related research (before, during, or at the end), and 11 said adolescents should be told at some point during research participation. Table 3 lists the detailed timing preferences of parents and caregivers regarding disclosure within the research context.

**Table 3:** Parents and caregivers' (n = 15) preferences on the timing of HIV disclosure to youth participating in HIV-related research

Timing	Disclosure to:	
	
	Children	Adolescents
Before participation	0	1

During enrolment	2	3

During research participation	2	3

At end of research; after research	2	2

Doctors should decide timing (but child or adolescent can be told)	2	2

Minors should not be told during the research	7	4

Parents and caregivers who suggested that children and/or adolescents should be told during the research or either at the end or after the research was completed explained that participation in the research would prepare the minor to learn her or his HIV-positive status.

For example, a maternal aunt and primary caregiver of a 12-year-old-boy, supported disclosure during study participation because:

The fact of participating will allow him to understand the study itself, [and] then his state of health.

Another caregiver, the maternal aunt and primary caregiver of a 12-year-old girl, described a similar rationale for supporting disclosure at the end of a study:

Their participation in the study will serve as a preliminary stage; they will discover themselves little by little while avoiding all trouble due to surprises. At the end of the study, the child will be well equipped to manage their situation [his or her HIV infection].

#### Health professional responses

Health professionals described their perceptions on the timing of disclosure of HIV status to minors in the context of HIV-related research participation, distinguishing between children and adolescents. Ten said that children should not be told their HIV-positive status at all during the research. Many believed that children would either not understand or would need time to be prepared before disclosure.

One health professional, a male social assistant, felt that this would discourage participation once enrolled:

It would not be desirable to tell them during the study. It can create frustrations. The child in question will not follow [instructions] very well or will stop participating.

In contrast, three health professionals felt that children should be told at the beginning of the research. Their reasons focused on reducing the children being taken aback upon learning their status during the course of the research and for enhancing the children's abilities to answer questions posed by study staff during the research.

For example, a male physician said:

It's better for the child to know before the study, so that there aren't problems: so that the child isn't surprised during the study for learning that he/she is seropositive. It won't be fair. It's good for him/her to know it beforehand.

Another health professional, a female agency leader, said:

[The child should be told] maybe when registering for the study – so, before he starts the study. You should tell them so that they can answer questions they will ask them, because people need information related to their status. It will be necessary that they know; if they know, they can answer in a better way.

Four health professionals believed that children should not be told at enrolment, but sometime during the course of the research or at the end of the research. Some health professionals also commented that the timing of disclosure depended on the type of study:

According to a male physician:

Well, it depends on the study, what is to be done. If it is part of the study or if during the study the child could be led to understand that he/she is seropositive – it's better for the child to know it before the study, so that there aren't problems: ... so that the child isn't surprised during the study for learning that he/she is seropositive.

Perceptions on when to disclose to adolescents in HIV-related research shifted slightly; fewer health professionals (n = 8) suggested that adolescents should not be told at all. Four health professionals believed that adolescents should be told at the beginning of the research, and five suggested disclosure take place during the research or at the end. Reasons provided were similar to those provided for children. Table 4 lists the detailed timing preferences of health professionals regarding disclosure within the research context.

**Table 4:** Health professionals' (n = 17) preferences on the timing of HIV disclosure to youth participating in HIV-related research

Timing	Disclosure to:	
	
	Children	Adolescents
Beginning of research participation	3	4

During research participation or at the end	4	5

Minors should not be told during the research	10	8

## Discussion

Within the context of participation in HIV-related research, youth perceived disclosure as necessary, while parents and caregivers' views and health professionals' views varied greatly. Among parents and caregivers, perceptions varied based on the age of the minor, with more believing that adolescents should be informed at some point during research participation compared to children. Similar beliefs were shared by health professionals working with HIV-infected youth.

Some parents and caregivers and health professionals believed disclosure should happen at the beginning of the research, but more believed disclosure should come during or toward the end of the research as participating in research could prepare the minor to learn her or his HIV-positive status. Many parents and caregivers and health professionals suggested, however, that minors do not need to know their HIV-positive status in order to participate in HIV-related research.

While parents and caregivers and youth were not asked to describe their understanding of research, it is reasonable to assume, based on their responses, that research may have been perceived by many to be longitudinal in nature rather than a one-time event. Additionally, all participants were recruited either from medical care facilities or from a psychosocial support programme and are believed to be a "research-naïve" population. For these reasons, it is possible that some participants may not have fully understood the differences between research and medical care in situations where research recruits from, or is perceived to be carried out in a medical setting. Many participants also implied that questionnaires would be administered to participants as part of the study, and may have perceived research as similar to our formative study or other types of research that include direct questions on participant experiences. We incorporated these assumptions into our recommendations.

Disclosure outside of the research context, particularly when treatment is involved, was perceived as necessary among youth, parents and caregivers and health professionals as a means to improve adherence to medication. This is echoed in other literature on disclosure of HIV status to infected minors in similar settings [10]. This is an important finding for supporting disclosure to youth participating in HIV-related research that provides treatment.

The ages in which youth were disclosed align with ages where assent may be obtained from minors. Further, most parents and caregivers did not regret their child learning her or his positive HIV status, and youth said it was better to be informed. These findings provide some support for incorporating disclosure or planning for disclosure into assent or research participation procedures.

Taking into consideration the varied perceptions of parents and caregivers and youth in this current study, the data do not support a conclusion that disclosure should always occur, or that it should never occur, as part of the assent process or research participation. Thus, to balance the wishes of children to be informed of an illness from which they are suffering and the hesitations and concerns regarding disclosure in a research context among parents and caregivers, we recommend that researchers consider the following when conducting HIV-related research with minors in settings where it is common that children with HIV do not know their status:

• Prior knowledge of HIV status among minors or disclosure of HIV status to minors should not be a mandatory component of any assent or informational process prior to minors joining HIV-related research, including research that involves treatment. Systems should be in place to assess whether the child knows her or his HIV positive status.

• The word, "HIV", should not be included when first explaining HIV-related research to minors unaware of their HIV infection. HIV should not be mentioned on the assent form or on the informational sheets about the research. Ethical committees reviewing such research should be apprised of the most current data surrounding HIV disclosure and assent in the study setting.

• When the HIV-related research involves the provision of treatment, it may be important to disclose HIV-positive status to minors as early as possible as a means to enhance adherence to medication. However, given the different perceptions of parents and caregivers on the timing of disclosure during research participation found in our study, care must be taken on determining when to disclose.

• When the HIV-related research does not involve the provision of treatment, researchers should take into consideration the type, objectives and length of the HIV-related research when deciding whether or not disclosure should be a component of the research, being mindful that the minors participating in the research may in fact want to know that they have HIV. Participation in the research could serve as a first step in the disclosure process, or planning for disclosure could be incorporated into study procedures.

• Given that minors in this research suggested that minors would want to know their HIV-positive status, an individualized disclosure plan should be initiated with parents or caregivers at the time of their child's enrolment in HIV-related research. For those parents or caregivers who are not ready to discuss disclosure at enrolment, the disclosure plan can include no disclosure at enrolment, but should incorporate a component to revisit the issue with parents or caregivers at a future date. This is especially important if the HIV-related research involves treatment.

• Where no national guidelines exist for assent procedures or disclosure to HIV-infected children, key stakeholders should develop such guidelines, including a process of community engagement, to ensure cultural appropriateness and compatibility with laws applicable to the rights of children.

Limitations of the study include its small sample size. Additionally, while interviewers were instructed to provide a definition and examples of research among HIV-positive youth as part of the interviews, we did not assess parents and caregivers' or youths' understanding of research. Participants' answers may have varied based on the type of research they envisioned (e.g., research involving only in-depth interviews versus clinical research on treatment) or their perceptions of research in general.

Research to compare attitudes toward disclosure among minors participating in different types of HIV research would have been an interesting extension of the study; however we did not design this study to answer this question. In addition, the parents and caregivers and youth who took part in this study may have not had enough prior exposure to research to be an appropriate population for such a study.

## Conclusion

The formative research findings confirmed that the approach we were using for obtaining assent from minors for our operational research was appropriate. Our findings also informed our programme guidelines that now recommend that discussions between clinic staff and parents or caregivers on disclosure to the minor begin at enrolment. The parental consent form used in our research has been updated to specifically mention that clinic staff will work with parents and caregivers to facilitate the HIV disclosure process and full disclosure by the child's 18^th ^birthday at the latest in order to enhance the child's adherence to treatment and reduce his or her risk of infecting others through sexual contact.

Researchers should consider omitting "HIV" when explaining HIV-related research to youth living with HIV, as well as excluding "HIV" in assent forms and informational sheets in settings where most minors are unaware of their HIV infection. Instead, an individualized disclosure plan should be initiated with parents or caregivers at the time of enrolment of youth in HIV-related research, particularly in research that involves treatment.

## Competing interests

The authors declare that they have no competing interests.

## Authors' contributions

AC, LV, JD, SR and FB conceptualized and designed the study. AC, LV, JD and SO undertook analysis and interpretation of data. All authors critically revised the article for intellectual content, and all approved the final version to be published.

## Authors' informations

AC and LV: are currently at Family Health International, Research Triangle Park, US. SO is currently with World Wildlife Fund, Kinshasa, Democratic Republic of the Congo.
